# Bacterial bloodstream infections in a tertiary infectious diseases hospital in Northern Vietnam: aetiology, drug resistance, and treatment outcome

**DOI:** 10.1186/s12879-017-2582-7

**Published:** 2017-07-12

**Authors:** Vu Quoc Dat, Hieu Ngoc Vu, Hung Nguyen The, Hoa Thi Nguyen, Long Bao Hoang, Dung Vu Tien Viet, Chi Linh Bui, Kinh Van Nguyen, Trung Vu Nguyen, Dao Tuyet Trinh, Alessandro Torre, H. Rogier van Doorn, Behzad Nadjm, Heiman F.L. Wertheim

**Affiliations:** 1 Department of Infectious Diseases, Hanoi Medical University, Hanoi, Vietnam; 2 National Hospital for Tropical Diseases, Hanoi, Vietnam; 3 Wellcome Trust Major Overseas Programme, Oxford University Clinical Research Unit, Hanoi, Vietnam; 4 Nuffield Department of Clinical Medicine, Centre for Tropical Medicine, University of Oxford, Oxford, UK; 5 Department of Medical Microbiology, Radboudumc, Nijmegen, Netherlands

**Keywords:** bacteremia, bloodstream infection, Vietnam, sepsis, drug resistance, bacterial, Gram-negative bacteria, *Streptococcus suis*, *Burkholderia pseudomallei*

## Abstract

**Background:**

Bloodstream infections (BSIs) are associated with high morbidity and mortality worldwide. However their aetiology, antimicrobial susceptibilities and associated outcomes differ between developed and developing countries. Systematic data from Vietnam are scarce. Here we present aetiologic data on BSI in adults admitted to a large tertiary referral hospital for infectious diseases in Hanoi, Vietnam.

**Methods:**

A retrospective study was conducted at the National Hospital for Tropical Diseases between January 2011 and December 2013. Cases of BSI were determined from records in the microbiology department. Case records were obtained where possible and clinical findings, treatment and outcome were recorded. BSI were classified as community acquired if the blood sample was drawn ≤48 h after hospitalization or hospital acquired if >48 h.

**Results:**

A total of 738 patients with BSI were included for microbiological analysis. The predominant pathogens were: *Klebsiella pneumoniae* (17.5%), *Escherichia coli* (17.3%), *Staphylococcus aureus* (14.9%), *Stenotrophomonas maltophilia* (9.6%) and *Streptococcus suis* (7.6%). The overall proportion of extended spectrum beta-lactamase (ESBL) production among *Enterobacteriaceae* was 25.1% (67/267 isolates) and of methicillin-resistance in *S. aureus* (MRSA) 37% (40/108). Clinical data was retrieved for 477 (64.6%) patients; median age was 48 years (IQR 36–60) with 27.7% female. The overall case fatality rate was 28.9% and the highest case fatality was associated with *Enterobacteriaceae* BSI (34.7%) which accounted for 61.6% of all BSI fatalities.

**Conclusions:**

*Enterobacteriaceae* (predominantly *K. pneumoniae* and *E. coli*) are the most common cause of both community and hospital acquired bloodstream infections in a tertiary referral clinic in northern Vietnam.

## Background

Bloodstream infections (BSIs) are an important cause of sepsis-related morbidity and mortality worldwide [1]. A systematic review of BSI burden in population based studies estimated the annual numbers of BSI episodes ranged from 575.462–677.389 in North America and 1,213,460–1,381,590 in Europe with the annual numbers of BSI associated deaths were 79,466–93,655 and 57,750–276,318 respectively [2]. In developed setting, the BSI and BSI associated septic shock still accounted for 6% and 3% of all admission to ICUs with the in-hospital mortality rates of 40% and 49% respectively [3]. Additionally, BSIs are associated with long-term risk of mortality among survivors [4]. In a population based study in Denmark, while the incidence of community and hospital acquired BSIs annually decreased 3.7% and 4.2% respectively between 1998 to 2008, the incidence rate of healthcare-associated bloodstream infection BSI remained unchanged [4]. Data from low and middle income countries (LMIC) on the causes of bloodstream infection are limited [5], but crucial for enabling clinicians to direct antimicrobial therapy appropriately and for the design of preventive measures. Useful lessons can be learned from analysing the bacterial aetiology of BSIs as most cases reflect severe illness and bacteria detected are usually directly responsible for disease [6, 7]. The causative agent will be affected by a number of factors, particularly the focus of infection, comorbidities like immunodeficiency, chronic renal and liver disease, in addition to socio-economic, climatic and other geographical factors [8]. The level of economic development can also impact aetiology distribution through changing exposure to environmental organisms associated with urbanisation, changing farming and food consumption practices, vaccination and comorbidities associated with increased wealth and improved life expectancy [9]. Consequently, data from tropical emerging economies are important to understanding and updating worldwide trends of the aetiology of severe bacterial disease in the tropics.

Vietnam is a low middle income country (LMIC), an emerging economy and a hotspot for emerging infectious diseases [10]. Levels of antibiotic resistance, an important cause of treatment failure and subsequent mortality are also high in Vietnam [11, 12]. In the Asian Network for Surveillance of Resistant Pathogens with 11 participating countries in 2001, Vietnam had a highest prevalence of pneumococcal multidrug resistance (defined as resistance to at least three classes of antibiotics) (71.4%) which much higher than the second and third highest countries and territories of Korea (54.8%) and Hong Kong (43.2%) [12]*.* In another study in 20 hospitals in 5 Asia-Pacific countries, the prevalence of carbapenem non-susceptible Gram-negative pathogens was highest at 35.0% in Vietnam, in which the prevalence of carbapenem non-susceptiblity among *Pseudomonas aeruginosa*, *Enterobacteriaceae* and *Acinetobacter baumannii* were 46.7%, 5.6% and 89.5% respectively [11].

In-hospital mortality among patients with sepsis is reduced by early initiation of adequate empiric antibiotic therapy [13, 14] and strategies to promote appropriate antibiotic use may help to prevent antimicrobial resistance and optimise use of hospital resources. However, the epidemiology of BSI in Vietnamese adults and associated treatment outcomes are not well studied. An understanding of BSI in Vietnam, its resistance patterns and their impact on patient’s outcomes is important to guide clinical management and appropriate antibiotic use. This article reports on a retrospective analysis of three years of BSI data at the National Hospital for Tropical Diseases (NHTD), a large tertiary referral hospital for infectious diseases in Hanoi, Vietnam.

## Methods

### Study design and data collection

This is a retrospective, cohort study of patients with bacterial bloodstream infection (BSI) admitted to the National Hospital for Tropical Diseases (NHTD) between January 1st, 2011 and December 31st, 2013. NHTD is a 300-bed tertiary teaching hospital that provides care to individuals suspected of an infectious disease living within its catchment area. NHTD is also a referral hospital assigned to all provincial hospitals in the north of Vietnam for infectious diseases that cannot be managed locally. As a result the hospital receives patients both directly from the community and transferred from hospitals in the region. The study included all positive blood cultures with recognized bacterial pathogens among patients who were hospitalized during the study period and additional clinical information from a subgroup of patients whose patient records were available to the investigators. A standard data collection form was created to extract demographic & clinical data, microbiology and outcome information from routine patient and microbiology records. Where patients had been transferred from another hospital, the transfer letter was examined for any record of antibiotic therapy having been provided at the referring hospital.

### Study definitions

A recognised bacterial pathogen identified as an organism which is not on the CDC’s National Healthcare Safety Network (NHSN) common commensal list unless there were 2 bottles from the same set were positive for the same organism or a single positive bottle plus an evident focus of infection (abscess or valvular vegetation). An episode of BSI was defined as the isolation of the organism(s) from one or more positive blood bottles which were explainable by a common source of infection and the time between two isolations was not interrupted by an asymptomatic period. When the clinical information was not available for judgment, the same organism(s) isolated within 48 h of another positive culture was considered as the same episode [15].

A case of community-acquired BSI (CABSI) was defined as a patient with a positive blood culture from a blood sample drawn ≤48 h after hospitalization at any health care facility (to ensure correct categorisation of patients referred from another hospital) for the current illness. Hospital-acquired BSI (HABSI) cases were defined as patients with >48 h between admission at any health care facility and the blood being drawn using the referring letter when applicable [16, 17]. These data were only available from patients whose clinical records were available. Any BSI caused by *Streptococcus suis* or *Burkholderia pseudomallei*, was classified as CABSI regardless of when samples were taken with the assumption that the acquisition of those infection due to occupational exposure to pigs and environmental exposure to contaminated environment are unlikely in the hospital setting respectively [18, 19].

Blood for culture, approximately 5–10 mL of blood, was collected in a single commercially sourced aerobic bottle and incubated in an automated system (Bactec, Becton Dickinson, USA) for five days (5–10 mL blood). Bottles that flagged positive were Gram-stained, subcultured and identified using standard microbiological techniques, including VITEK and API testing (bioMérieux, France). Antibiotic susceptibilities were performed using CLSI guidelines with breakpoints from 2013 [20]. For *S. suis*, the susceptibility was evaluated using CLSI breakpoints for *Streptococcus viridans* group [21].

Fatal cases were defined as patients who died in hospital or were discharged with diagnosis of brain death and the expectation that they will die imminently within hours or days (palliative discharge to die at home, a common practice in Vietnam).

### Data entry and statistical analysis

Data were entered in Epidata (EpiData Association, Odense, Denmark) and analysed using SPSS 22.0.0 (IBM Co., Armonk, NY, USA). Standard descriptive statistics were calculated for categorical (in percentage) and continuous variables (median and interquartile, IQR). Bivariate analyses were performed using Pearson’s chi-squared test or Fisher’s Exact Test for categorical variables as appropriate. The possible selection bias was assessed by chi-square and t-test analyses to compare gender and age differences between those with and without clinical outcomes, respectively. Cox proportional-hazards regression was used to identify variables that predict clinical outcomes. Cox’s proportional hazards regression was performed to analyse factors associated with all-cause in-hospital mortality. BSI episodes of polymicrobial infection and *S. maltophilia* infections were excluded from regression analysis due to a small number and an uncertain pathogenicity respectively. In cases where there were multiple blood cultures positive, we only included the first positive blood culture. All tests were two-tailed and differences were considered statistically significant at *p* values ≤0.05.

## Results

### Study population’s demographics

**Fig. 1:**
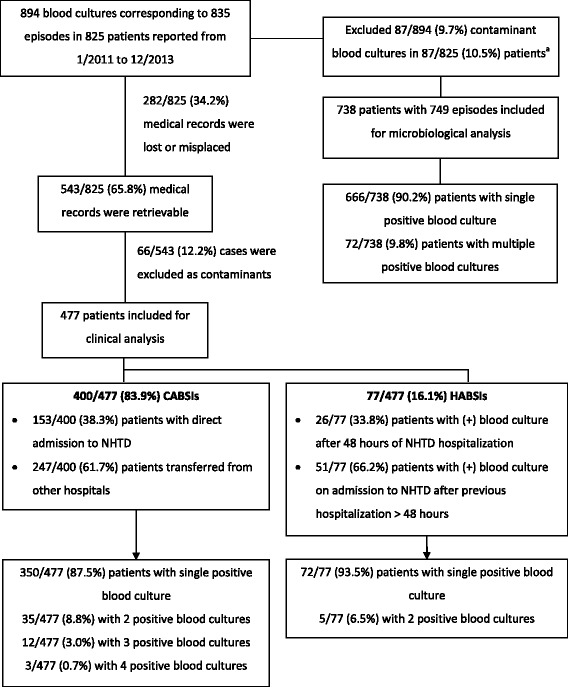
Flowchart of BSI episodes and classification of BSI by location of acquisition. Classification based on the first isolate during hospitalization. HABSI: Hospital-acquired bloodstream infection; CABSI: Community-acquired bloodstream infection. **a** Contaminant episodes included 22 episodes of *B. cepacia*, 25 episodes of *Alcaligenes spp.*, 7 episodes of viridans group streptococci, 8 episodes of *Ralstonia picketii*, 6 episodes of Pseudomonas species (*P. fluorescens, P. putida* and *P. stutzeri*) 4 episodes of *Chryseobacterium spp., 3 episodes of S. maltophilia, 3 episodes of Serratia marcescens,* 3 episodes of *Ochrobactrum antrhopi,* 2 episodes of *Sphingomonas paucimobilis,* 1 episode of each *E. coli, K. pneumonia, Corynebacterium spp.* and *Chryseomonas luteola*

There were 894 positive blood cultures corresponding to 835 BSI episodes in 825 patients recorded between January 2011 and December 2013. Notes from 543/825 (65.8%) patients could be retrieved to collect clinical details of individual patients while 282 (34.2%) were not available (Fig. 1). Sensitivity analysis of age and gender showed no differences between cases with and without clinical outcome (chi-square test, *p* = 0.664 and t-test, *p* = 0.053 respectively). Among collected records, sixty-six cases (12.2%, 66/543) were subsequently classified by cultured organism as having a pseudo-bacteremia due to contamination, including 16 cases with *Burkholderia cepacia,* 19 cases with *Alcaligenes species,* 5 cases with *Ralstonia pickettii* and 4 cases with *Pseudomonas species (*2 *P. putida* and 2 *P. stutzeri)*. There were 3 cases with *Serratia marcescens*, 3 *S. maltophilia*, 1 *K. pneumoniae* and 1 *E. coli* which were classified as contaminants by treating doctors at the time of results return, based on patient’s survival and recovery without antibiotic treatment for bacterial disease and with an alternative possible viral diagnosis. Other contaminated cultures were 3 cases with *Ochrobactrum anthropi*, 1 case with *Chryseobacterium indologenes*, 1 case with *Chryseomonas luteola*, 1 case with (non-diphtheroid) *Corynebacterium spp.*, 1 case with *Staphylococcus spp.* and 7 cases with single culture of viridans group streptococci without an endovascular or other focus. As a result, 477 cases were included for the clinical analysis.

The median age of the group of 477 patients with BSI was 48 years (IQR 36–60), 27.7% of patients were female (132/477), 14 (10.6%) of whom were pregnant). Common co-morbidities were chronic hepatitis or cirrhosis (14.3%, 68/477), diabetes mellitus (7.1%, 34/477) and HIV infection (6.3%, 30/477) (Table 1).

**Table 1:** Characteristics of the clinical study population

	Total	Community acquired BSI	Hospital acquired BSI
	*N* = 477	*N* = 400	*N* = 77
		*n* (%)	*n* (%)
Age – median (IQR)	48 (36–60)	48 (36–60)	50 (32–60)
Female sex	132 (27.7%)	122 (30.6%)	10 (13%)
Any ICU admission during NHTD hospitalisation	245 (51.4%)	190 (47.5%)	55 (71.4%)
Any history of medical condition	142 (29.8%)	109 (27.3%)	33 (42.9%)
Liver disease	68 (14.3%)	54 (13.5%)	14 (18.2)
Diabetes	34 (7.1%)	25 (6.3%)	9 (11.7%)
Moderate or severe renal disease	9 (1.9%)	6 (1.5%)	3(3.9%)
HIV infection	30 (6.3%)	21 (5.3%)	9 (11.7%)
Other morbidity	10 (2.1%)	8 (2%)	2 (2.6%)
Long-term corticosteroid use	8 (1.7%)	7 (1.8%)	1 (1.3%)
Self-report alcoholism	67(14%)	56 (14%)	11 (14.3%)
Intravenous drug use	23 (4.8%)	17 (4.3%)	6 (7.8%)

^a^Liver disease was defined in a patient with chronic hepatitis and/or cirrhosis

^b^Moderate or severe renal disease was defined in a patient with baseline creatinine >3 mg% (265 umol/l), dialysis or kidney transplantation

In patients with CABSIs, 12.5% (50/400) had multiple positive blood cultures with either *S. aureus* or *K. pneumoniae*. Whilst most patients with CABSI were admitted directly from the community, 153/400 patients (38.3%) were transferred from other hospitals within 48 h, with 62/153 patients (40.5%) recorded as having received antibiotics before admission to the study hospital.

### Aetiology of bloodstream infections

#### Analysis of microbiological dataset

During the 3 year period, a total of 738 BSI patients were identified with 67.9% (501/738) with Gram-negative bacteria isolated, 31% (229/738) Gram-positive bacteria, and 1.1% (8/738) had two pathogens isolated in the same culture bottle (*n* = 8). *Enterobacteriaceae* were the most frequently isolated group of organisms among patients with BSI (46.7%, 345/738) with the predominant *Enterobacteriaceae* being *K. pneumonia*e (37.4%, 129/345) and *E. coli* (37.1%, 128/345), followed by *Salmonella* enterica (13.0% or 45/345) (Table 2).

**Table 2:** Aetiology of bloodstream infections

Pathogen	All patients	Sampled clinical cases			
		Total	CABSI	HABSI	p^g^
	*N* = 738	*N* = 477	*N* = 400	*N* = 77	
Enterobacteriaceae	345 (46.7%)	245 (51.4%)	200 (50%)	45 (58.4%)	0.213
*Klebsiella pneumoniae*	129 (17.5%)	110 (23.1%)	88 (22%)	22 (28.6%)	0.237^d^
*Escherichia coli*	128 (17.3%)	73 (15.3%)	64 (16%)	9 (11.7%)	0.391
*Salmonella* enterica	45 (6.1%)	30 (6.3%)	24 (6%)	6 (7.8%)	0.606
serovar Typhi	10 (1.4%)	4 (0.8%)	4 (1.00%)	0	1
non-typhi Salmonella	35 (4.7%)	26 (5.5%)	20 (5%)	6 (7.8%)	0.407
*Serratia marcescens*	16 (2.2%)	12 (2.5%)	9 (2.3%)	3 (3.9%)	0.421
*Enterobacter* species	16 (2.2%)	12 (2.5%)	8 (2.0%)	4 (5.2%)	0.112
Other *Enterobacteriaceae* species^a^	11 (1.5%)	8 (1.7%)	7 (1.8%)	1 (1.3%)	1
Non-Enterobacteriacae	156 (21.1%)	99 (20.8%)	78 (19.5%)	21 (27.3%)	0.127
*Stenotrophomonas maltophilia*	71 (9.6%)	47 (9.9%)	39 (9.8%)	8 (10.4%)	0.836
*Aeromonas* species	34 (4.6%)	14 (2.9%)	12 (3.0%)	2 (2.6%)	1
*Burkholderia pseudomallei*	15 (2.0%)	13 (2.7%)	13 (3.3%)	0	
*Pseudomonas aeruginosa*	16 (2.2%)	13 (2.7%)	9 (2.3%)	4 (5.2%)	0.240
*Acinetobacter* species^b^	16 (2.2%)	10 (2.1%)	3 (0.8%)	7 (9.1%)	<0.001
*Other gram-negative bacteria* ^*c*^	4 (0.5%)	2 (0.4%)	2 (0.5%)	0	1
Gram-positive isolates	229 (31.0%)	126 (26.4%)	115 (28.8%)	11 (14.3%)	0.007
*Staphylococcus aureus*	110 (14.9%)	41 (8.6%)	34 (8.5%)	7 (9.1%)	0.826
*Streptococcus suis*	56 (7.6%)	43 (9.0%)	43 (10.8%)	0	-
*Streptococcus* species	37 (4.9%)	23 (4.8%)	21 (5.3%)	2 (2.6%)	0.559
*S. pneumoniae*	7 (0.9%)	5 (1.0%)	4 (1.0%)	1 (1.3%)	0.587
*S.* viridans group^d^	21 (2.8%)	11 (2.3%)^b^	10 (2.5%)	1 (1.3%)	1
*Beta-hemolytic streptococci*	9 (1.2%)	7 (1.5%)	7 (1.8%)	0	0.604
*Enterococcus* species	14 (1.9%)	12 (2.5%)	10 (2.5%)	2 (2.6%)	1
*Other gram-positive bacteria* ^*e*^	12 (1.6%)	7 (1.5%)	7 (1.8%)	0	0.604
Polymicrobial^f^	8 (1.1%)	7 (1.5%)	7 (1.8%)	0	0.604

^a^Including 3 cases with *Klebsiella oxytoca*, 3 cases with *Proteus mirabilis*, 2 cases with *Escherichia hermannii,* 1 case with *Citrobacter freundii*, 1 case with *Morganella morganii and 1 case with Vibrio vulnificus*

^b^Including 12 cases with *A. baumannii*, 3 cases with *A. lwoffii* and 1 case with *A. junii*

^c^Including 1 case for *Elizabethkingia meningosepticum*, *Haemophilus influenzae*, *Pasteurella multocida* and *Neisseria meningitidis*,

^d^Cases with viridans group infection had at least two positive blood culture or single positive blood culture plus vegetation on echocardiography

^e^Including 4 cases with *Rhodococcus equi*, 3 cases with *Listeria monocytogenes*, 2 cases with *Aerococcus viridians*, 2 cases with *Listeria spp.* and 1 case with *Listeria innocua*

^f^In 8 dual infection cases, 3 HIV infected cases had co-infection with *Talaromyces marneffei* and *S. aureus*, *Escherichia hermannii* or *Salmonella group D*; 1 case with *S. aureus* and *K. pneumoniae*, 1 case with *Enterococcus faecalis* and viridans streptococci, 1 case with *E. coli* and *K. pneumoniae* and 1 case with *S. aureus* and *E. coli* co-infection

^g^ *p* values were calculated using Chi-square test or Fisher’s Exact test as appropriate

Among Gram-positive isolates, *S. aureus* was the leading pathogen (48% or 110/229), followed by *S. suis* (24.5% or 56/229) and other *Streptococcus* species (16.2% or 37/229). Non- *Enterobacteriaceae* Gram-negative bacteria were identified in 156 cases (21.1%), of which *S. maltophilia* accounted for 45.5% (71/156).

The overall proportion of extended spectrum beta-lactamases (ESBL) production among *Enterobacteriaceae* was 25.1% (67/267 isolates, 78 missing result). Tables 3 and 4 display the antibiotic resistance of *K. pneumoniae* and *E. coli*. The prevalence of ESBL production among *E. coli* isolates was significantly higher than in *K. pneumoniae* (45%, 50/111 vs 12.3%,15/122, *p* < 0.001). Among Gram-positive bacteria, 97.4% (38/39) of *S. suis* isolates were susceptible to penicillin with penicillin MIC median of 0.032 (IQR 0.023–0.032) and only 1 case was intermediate susceptible to penicillin with an MIC of 0.23, though no confirmatory testing was performed. Methicillin resistance was observed in 37% (40/108) of *S. aureus* isolates and all 29 MRSA isolates tested with vancomycin E-test were susceptible. Among 67 *S. aureus* isolates tested for vancomycin by E-test, 62.7% (42/67) of isolates had vancomycin MIC ≤1 μg/ml and 37.3% (25/67) 1.5–2 μg/ml (Table 5).

**Table 3:** Resistance among *K. pneumoniae* from BSI

Resistance	All isolates	Sampled clinical cases			
		Total	CABSI	HABSI	*P* value ^a^
Any extended spectrum cephalosporin	19/133 (14.3%)	13/112 (11.6%)	8/90 (8.9%)	5/22 (22.7%)	0.128
ESBL production	15/122 (12.3%)	9/101 (8.9%)	5/82 (6.1%)	4/19 (21.1%)	0.062
Others	4/133 (3.0%)	4/112 (3.6%)	3/90 (3.3%)	1/22 (4.5%)	1
Any carbapenem	1/131 (0.8%)	1/109 (0.9%)	0/89 (0%)	1/20 (5%)	0.183
Any aminoglycoside	17/131 (13.0%)	13/109 (11.9%)	9/89 (10.1%)	4/20 (20%)	0.252
Any fluoroquinolone	11/131 (8.4%)	8/109 (7.3%)	4/89 (4.5%)	4/20 (20%)	0.036
Co-trimoxazole	24/124 (19.4%)	19/102 (18.6%)	14/84 (16.7%)	5/18 (27.8%)	0.318

^a^ *p* values were calculated using Fisher’s Exact Test

**Table 4:** Resistance among *E. coli* from BSI

Resistance	All isolates	Sampled clinical cases			
		Total	CABSI	HABSI	*P* value^a^
Any extended spectrum cephalosporin	72/131 (55.0%)	45/74 (60.8%)	39/65 (60%)	6/9 (66.7%)	1
ESBL production	50/111 (45%)	33/61 (54.1%)	28/52 (53.8%)	5/9 (55.6%)	1
Others	22/131 (16.8%)	12/74 (16.2%)	11/65 (16.9%)	1/9 (11.1%)	1
Any carbapenem	1/130 (0.8%)	0/73 (0.0%)	0/64 (0.0%)	0/9 (0.0%)	-
Any aminoglycoside	30/129 (23.3%)	15/73 (20.5%)	14/64 (21.9%)	1/9 (11.1%)	0.675
Any fluoroquinolone	41/129 (31.8%)	26/72 (36.1%)	22/63 (34.9%)	4/9 (44.4%)	0.714
Cotrimoxazole	62/90 (68.9%)	27/38 (71.1%)	24/33 (72.7%)	3/5 (60.0%)	0.615
Fosfomycin	1/52 (1.9%)	1/44 (2.3%)	1/38 (2.6%)	0/6 (0%)	-

^a^ *p* values were calculated using Fisher’s Exact Test

**Table 5:** Resistance among *S. aureus* from BSI

Susceptibility	All isolates	Sampled clinical cases			
		Total	CABSI	HABSI	*P* value ^a^
Methicillin (MRSA)	40/108 (37%)	12/38 (31.6%)	12/31 (38.7%)	0/7 (0%)	0.074
Erythromycin	39/72 (54.2%)	11/23 (47.8%)	10/18 (55.6%)	1/5 (20%)	0.317
Clindamycin	56/109 (51.4%)	21/40 (52.5%)	18/23 (54.5%)	3/7 (42.9%)	0.689
Chloramphenicol	35/105 (33.3%)	14/38 (36.8%)	11/31 (35.5%)	3/7 (42.9%)	1
Gentamicin	18/76 (23.7%)	9/26 (34.6%)	7/21 (33.3%)	2/5 (40%)	1
Ciprofloxacin	19/99 (19.2%)	7/33 (21.2%)	6/28 (21.4%)	1/5(20%)	1
Levofloxacin	18/103 (17.5%)	9/40 (22.5%)	6/13 (18.2%)	3/7 (42.9%)	0.316
Co-trimoxazole	8/62 (12.9%)	5/28(17.9%)	4/21 (19%)	1/7 (14.3%)	1
Rifampicin	10/95 (10.5%)	6/33 (18.2%)	5/27 (18.5%)	1/6 (16.7%)	1
Vancomycin^b^	0/63 (0%)	-	-	-	-

^a^ *p* values were calculated using Fisher’s Exact Test

^b^The vancomycin susceptibility and the MRSA rate were defined by Minimum Inhibitory Concentrations (MICs) using E-test

### Analysis of the clinical dataset

The proportions of CABSI and HABSI caused by *Enterobacteriaceae* were not statistically different (55.1%, 203/397 vs 58.4%, 45/77, *p* = 0.24), similar for for non-enterobacteriaceae Gram-negative bacteria (19.6%, 78/397 vs 27.3%, 21/77, *p* = 0.132). In contrast, Gram-positive bacteria were more frequently identified in CABSI (29.2%, 116/397 vs 14.3%, 11/77, *p* = 0.011) and *Acinetobacter* species were more common in HABSI (Table 2).

There was no difference in the proportion of ESBL production in community versus hospital setting among *E. coli* isolates (53.8%, 28/52 vs 55.6%, 5/9, *p* = 1.0). In *K. pneumoniae* isolates, the proportion of ESBL production were higher in HABSI (21.1%, 4/19 vs 6.1%, 5/82 vs, *p* = 0.039). Compared with *K. pneumoniae*, *E. coli* isolates were also more frequently resistant to aminoglycosides (23.3%, 30/129 vs 13.0%, 17/131, *p* = 0.031) and fluoroquinolones (31.8%, 41/128, vs 8.4%, 11/131, *p* < 0.001).

*K. pneumoniae* isolates associated with HABSI more frequently showed antibiotic resistance compared to CABSI isolates, though statistical significance was only reached for quinolones, while the pattern of resistance among *E. coli* isolates did not differ between the hospital and community (Tables 3 and 4). MRSA rates differed between community (38.7%, 12/31) and hospital settings (0/7), though this did not reach statistical significance (*p* = 0.074). Case notes were not retrievable in 69/110 (62.7%) cases of *S. aureus* bacteremia.

### Case fatality of bloodstream infection

**Fig. 2:**
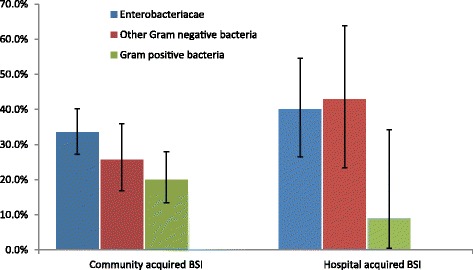
Case-fatality by etiology of bloodstream infection (BSI). Error bars represent the 95% confidence interval (CI) for case mortality proportions

Case fatality was assessed in the clinical dataset only. The overall in-hospital case fatality rate among patients was 138/477 (28.9%). Overall case fatality rates among patients infected with *Enterobacteriaceae*, non-*Enterobacteriaceae* and Gram-positive bacteria were 34.7% (85/245), 29.3% (29/99) and 19% (24/126), respectively (Fig. 2). There was no case of death among patients with mix infections (0/7). Case fatality rate in CABSI (27.5%, 110/400) was lower, but not statistically different from HABSI (36.4%, 28/77) (*p* = 0.131). In the most frequent isolates of *E. coli*, *K. pneumonia*e, *S. aureus*, *S. maltophilia* and *S. suis*, case-fatality rates were 35.6% (26/73), 35.5% (39/110), 26.8% (11/41), 17.0% (8/39) and 9.3% (4/39).

Multivariate Cox proportional hazard regression analysis was used to measure the effect of demographics, acquisition of infection, co-morbidities, previous hospital referring factors and bacterial species. Independent factors associated with case fatality were *Enterobacteriaceae* and non-*Enterobacteriaceae* infection (HR 2.485, 95%CI 1.546–3.994 and HR 1.931, 95% CI 1.063–3.50, respectively), co-morbidities (HR 1.467, 95% CI 1.017–2.114) and referring from other hospital (HR 2.049, 95% CI 1.411–2.975) (Table 6).

**Table 6:** Cox proportional hazards model of factors associated with all-cause in-hospital case fatality among patients with bloodstream infections due to a single organism in clinical dataset

Variable	Hazard ratio (95% CI)^a^	*P*-value^b^
Gender (male/female)	1.347 (.857–2.115)	0.196
Age (1-yr. increment)	1.008 (0.998–1.01)	0.118
Aetiology of BSI (gram-positive bacterial infection as reference)		
*Enterobacteriaceae* infections	2.485 (1.546–3.994)	<0.001
Non *Enterobacteriaceae* Gram-negative infections	1.931 (1.063–3.50)	0.031
Acquisition of infection (CABSI as reference)	1.006 (0.633–0.399)	0.053
Any co-morbidities	1.467 (1.017–2.114)	0.040
Referring from another hospital	2.049 (1.411–2.975)	<0.001

^a^Cases with *S. maltophilia* infection were excluded from analysis

^b^Hazard ratios and *P*-values were calculated using Cox proportional hazards model

## Discussion

This retrospective study includes 3-years of complete microbiological data on aetiology of BSI for 738 patients and almost two thirds (64.6%, 477/738) of these patients for clinical outcome analysis in a tertiary hospital in the north of Vietnam.

Our study shows the important role of pathogenic Gram-negative bacteria with a high proportion of cephalosporin-resistant *E. coli* and *K. pneumoniae* isolates and high prevalence of MRSA in CABSI. Our study shows the high contribution of *Enterobacteriaceae* BSI to mortality among adults admitted to our hospital in Vietnam, with over 60% of deaths associated with this group of bacteria. This study supports the observed shift within *Enterobacteriaceae* organisms from typhoidal and non-typhoidal *Salmonella* to *K. pneumoniae* and *E. coli*, the current leading causes of community acquired blood stream infections.

In Vietnam, *Salmonella enterica* serovar Typhi was reported to be the major cause of BSI, accounting for up to 74% of isolates from blood in the 1990s but gradually declined to 6% by 2008 [22]. With the recent emergence of *K. pneumoniae* and *E.coli*, we observed the high prevalence of extended spectrum Beta-lactamase (ESBL) production in these pathogens, especially in the hospital setting. In surveillance data from five Asia-Pacific countries in 2010, Vietnam reported the highest rate of ESBL positive *Enterobacteriaceae* isolates with an overall prevalence of 55.1% and 80.1% in ICU patients [11]. The high prevalence of antibiotic resistance in Vietnam may be attributed to multiple factors, such as dispensing practices in the community, lack of enforcement of regulation, limited resources for microbiological diagnostics, injudicious use of antibiotics in hospital, community and agriculture and insufficient antibiotic resistance surveillance and infection control [23].

In our study, ESBL production was detected among 6.1% *K. pneumoniae* from CABSI, which is consistent with the low ESBL prevalence of 4.1% among *K. pneumoniae* oropharyngeal carriers reported previously from northern Vietnam [24]. The rate of ESBL-production in *E. coli*(45%) was higher than in Thailand (CABSI, 11.8%) and Korea (31.3% in HABSI and 8.8% in CABSI) and comparable to the prevalence of ESBL among *E. coli* isolates in BSI in Cambodia (47.7%), [15, 17, 25]. The high rate of ESBL carriage in *E. coli* from CABSI is deeply worrying and likely reflects extensive overuse of oral cephalosporins in the community, their use in agriculture or other environmental sources [26, 27]. *S. suis* is a well-recognized zoonotic pathogen associated with bacterial meningitis and BSI among adults in Vietnam [10] and was noted as an important Gram-positive pathogen cultured from blood with high levels of susceptibility to penicillin.

Our study showed a higher case-fatality rate (110/400 or 27.5%) among community-acquired bacterial bloodstream infections than the rate of 9% reported in a review from 1990 to 2010 in south and southeast Asia and the rate of 18.1% among patients with CABSI in a meta-analysis in Africa from 1984 to 2006 [28, 29]. This difference is likely related to the changes in spectrum of aetiology with higher prevalence of *Salmonella spp.* (53.2% of *Enterobacteriaceae* isolates) in the past [28], the emergence of high levels of antibiotic resistance in Vietnam and the tertiary referral nature of the study hospital. The overall mortality in our study was slightly higher than the overall mortality of 22.5% in Cambodia where the epidemiology and pattern of resistance are similar [15]. We reported a high mortality of 35.6% among E. coli BSI, which is higher than mortality of 18.2% and 8% and in a large surveillance in England and in a population-based study in Finland [30, 31]. It is not clear about the reasons of the differences, but it may relate to pneumonia focus, severity of infection, antibiotic susceptibilities and seasonal variation [31]. As NHTD is a tertiary referral hospital, potentially more severe cases or cases that have failed therapy elsewhere may have been referred as compared to the hospitals included in other studies, leading to higher resistance and case fatality rates.

In North America and Europe, the nosocomial BSI case fatality rate ranges from 12 to 32% [2]. Changes in the aetiology of HABSI began in the 1960s–1970s in the United States of America (US) with replacement of Gram-negative by Gram-positive organisms, such that 65% of BSIs were *S. aureus*, the most common nosocomial pathogen [32]. However, a Vietnamese population based study indicated *S. aureus* nasopharyngeal carriage proportion (33.8%, CI 29.4–38.8) was higher than in European regions and the States [33], and *S. aureus* BSI is still mainly community-acquired in Vietnam [34]. In Vietnam the proportion of BSI attributable to *S. aureus* did not change during the 15-years from 1994 to 2008 [22]. Gram-negative bacilli are still recognized as the most frequent nosocomial isolates in developing countries [35]. In a multicenter surveillance study in Thailand, Gram-negative bacteria accounted for 67.6% of HABSI, of which *Acinetobacter spp.* (16.2%) was detected most frequently, followed by *K. pneumoniae* (13.9%) and *S. aureus* (13.9%) and the in-hospital mortality was 26.6% [36]. The overall case fatality rate of 36.4% among HABSI in our study population was higher than seen in North America and Europe. This may be related to higher levels of antibiotic resistance, differences in the availability of resources or the nature of admissions to NHTD, as described earlier.

We observed a predominance of males among patients with BSI (72.3%). This phenomenon was similar as in the cause of death reports in another tertiary hospital in the north of Vietnam where the top three of causes of death were septic shock, intracerebral haemorrhage and pneumonia. The proportion of males in this report was higher than females (1577/2340 or 67.4%) [37].

Among common isolates causing BSI, we reported the high prevalence of *S. maltophilia* (9.6%. 71/738) which is only rarely considered a pathogen, particularly from the community [32]. Whilst *S. maltophilia* was described as a recognised cause of CABSI among patients with comorbidities in a systematic review [38] and a recent publication from Taiwan [39], another publication describes contamination of blood culture bottles with *S. maltophilia* due to growth of the bacteria in disinfectant [40]. Furthermore, we have observed pseudoepidemics of *S. maltophilia* in this hospital (multiple patients from geographically disparate sites with different infection syndromes, newly admitted on the same ward with *S. maltophilia* cultured from blood at the same time). The retrospective nature of this study makes it difficult to define whether these isolates were true pathogens or represented contamination. Given how frequently this organism was isolated, further prospective investigation is required.

Our study has some important limitations. Firstly, NHTD is a tertiary referral hospital and specializes in infectious diseases. Therefore, the pattern of aetiology, resistance and spectrum of clinical disease is different from that seen in district and provincial level hospitals due to referral of certain syndromes, more severe and non-responsive cases.

Secondly, during the study period, only a single blood culture was routine collected and as a cost saving technique, routine laboratory practice was that cultures showing obvious contamination (multiple organisms, skin flora etc.) were discarded before full work up without recording this finding. This will have lowered estimations of blood culture sensitivity, which can be improved with more blood cultures and higher volumes of blood [41, 42]. Additionally, although the isolates of Enterobacteriaceae members are almost always interpreted as true bloodstream infections, single blood culture practice may underestimate the roles of fastidious pathogens in the era of increased catheters and prosthetic devices-related bloodstream infections [43].

In our study hospital acquired BSI was defined as cases with positive blood culture after any hospitalization for more than 48 h. Thus, patients who in reality had CABSI but with persistent bacteraemia, who were transferred after an inpatient stay at another hospital may have been misclassified as HABSI. The significance and role of certain bacteria, such as *S. maltophilia* or *Aeromonas* species, which have been associated with both pseudo-bacteraemia and real infection was not easy to establish because of a lack of repeat sampling in most of patients and the retrospective nature of the study. Lastly, clinical outcome analysis was only applied for 64.6% (477/738) of patients during the study period because the remaining patient’s notes could not be retrieved. This has resulted in significant gaps in the data and may have introduced bias if the sample was not random.

## Conclusions

Gram-negative bacteria are the most common cause of both community and hospital acquired blood stream infection in adults presenting to our tertiary referral hospital, with high associated mortality. The extended spectrum beta-lactamase (ESBL)-producing *Enterobacteriaceae* are prevalent in both community and health care setting. Our findings further support the need, as described in the GARP situation analysis [44] and the 2013 Vietnamese National Action Plan for AMR [45], for establishment of a comprehensive microbiological and antibiotic susceptibility surveillance system in Vietnam, with representation of the different levels of healthcare and to inform treatment guidelines and other public health measures.

## Abbreviations

BSIs: Bloodstream infections
CABSI: Community-acquired bloodstream infections
ESBL: Extended Spectrum Beta-Lactamases
HABSI: Hospital-acquired bloodstream infections
IQR: Interquartile range
LMIC: Low and middle income countries
MICs: Minimum Inhibitory Concentrations
MRSA: Methicillin-resistant *Staphylococcus aureus*
NHTD: National Hospital for Tropical Diseases
